# Gold Nanoparticles as Colorimetric Sensors for the Detection of DNA Bases and Related Compounds

**DOI:** 10.3390/molecules25122890

**Published:** 2020-06-23

**Authors:** Emilia Iglesias

**Affiliations:** Departamento de Química, Facultad de Ciencias, Campus A Zapateira, Universidade A Coruña, 15008 La Coruña, Spain; emilia.iglesias@udc.es

**Keywords:** biosensors, nucleobases, gold nanoparticles, colorimetric detection

## Abstract

Results regarding interaction of colloidal gold solutions with nucleobases, including uracil (U), as well as its sulfur derivatives, 2-thiouracil (2TU) and 4-thiouracil (4TU), cytosine (C), adenine (A), and guanine (G), as well as urea and thiourea (TU), are reported. Anionic stabilized citrate gold nanoparticles (AuNPs) were synthesized by reducing the tetrachloroaurate (III) trihydrate with trisodium citrate. The surface plasmon resonance (SPR) band was used in the characterization of synthesized AuNPs, as well as transmission electron microscope (TEM) imaging, which was used in the characterization of dispersed and aggregated gold nanoparticles. Interactions of nucleobases with the gold surface was analyzed by following the plasmon absorbance band red shift of the AuNPs. The sulfur-containing compounds adsorbed to the nanoparticle surfaces by chemisorption-type interactions; with TU and 4TU, the process is accompanied by a sudden change in color; in contrast, 2TU forms stable functionalized gold nanoparticles. Urea and U do not adsorb to nanoparticle surfaces, but the other heterocyclic bases containing nitrogen interact effectively with the gold surface, causing the assembly of nanoparticles, even though the interparticle self-aggregation process was slower than that mediated by either TU or 4TU. The method is efficient in the colorimetric detection of nucleobases and derivatives at concentration levels on the order of 1 µM.

## 1. Introduction

Interactions between DNA nucleobases and noble metallic surfaces are important for the exploitation of the biological reactivity used in biosensor development, nanoparticle-assisted cancer treatment, and DNA microarray optimization [1,2,3]. In recent years, colorimetric assays based on gold nanoparticles (AuNPs) have emerged as a promising new method due to their unique and size-dependent optical, electrical, and catalytic properties [4,5,6,7,8]. AuNPs exhibit a visible surface plasmon resonance (SPR) band, which gives them a singular red color in aqueous solution [9,10,11,12,13].

Compared to planar gold, dispersed AuNPs possess the advantage of maintaining a “clean” surface for a long time, larger surface area, and allow for faster reaction rates. Gold colloids are prepared and stored in aqueous solution, and, although the surface adsorbs certain compounds, such as citrate, which is used in the most common synthesis procedure to stabilized the gold clusters, these adsorbed ligands can be easily displaced by many other species with high reactivity towards gold. For example, thiol-containing molecules spontaneously attach to the gold nanoparticles’ surfaces through the formation of –S–Au bonds [14,15,16,17,18,19,20,21].

The properties of AuNPs are characterized not only by the properties of the metal cluster core, but also by those of the organic molecules that constitute the monolayer and contribute to cluster stabilization. In this sense, the plasmon frequency is sensitive not only to the nature of the capped gold molecules that determines its microenvironment, but also to the size of nanoparticles (NPs) [22]. Upon reduction of the interparticle distance, the SPR band can couple, and red-shift in the absorption spectrum is observed. Surface modifications of gold NPs using functionalized capping molecules has been exploited in the development of new diagnostic tools for biological compounds. DNA bases are the fundamental constituents of nucleic acids containing nitrogen found within nucleotides. The nucleic acids are the most studied biomolecules for capping gold NPs, and have been used with different goals that include the colorimetric detection of duplex and triplex DNA-binding molecules [2,6], DNA methylation as an early sign of tumor cells [6], or in gold nanoparticle-assisted cancer treatment [7,8].

The optical absorption spectrum of well-dispersed gold nanoparticles is widely used in colorimetric methods because, firstly, gold hydrosols have extremely high extinction coefficients, and thus very small concentrations (at the nM scale) are enough for visual observation, and secondly, upon aggregation of AuNPs, the color change is easily detected.

**Scheme 1:**
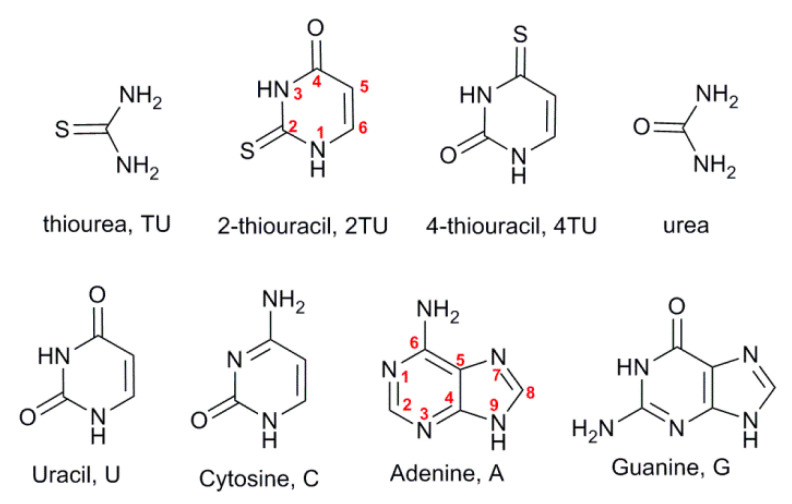
Molecular structures of the studied compounds.

In this study, we explore the interactions of dispersed gold hydrosols with the nucleobases uracil (U), cytosine (C), guanine (G), and adenine (A) along with other compounds of similar structure, such as urea, thiourea (TU), 2-thiouracil (2TU), and 4-thiouracil (4TU), see Scheme 1.

These compounds have different protonation and deprotonation sites. A considerable amount of work has been done in identifying these sites [23,24,25,26,27,28], in addition to the systematic studies examining the protomeric tautomerism of these heterocyclic compounds in their neutral and ionic forms [29,30]. Briefly, the pK_a_ values of the protonated forms (pK_a1_) of urea, thiourea, and uracil are reported as 0.05; −0.90, and <0.5, respectively [24]. In line with the increased acidity of sulfur derivatives, the pK_a1_ values of 2TU and 4TU should be appreciably lower than that of uracil (no data were found). The pK_a1_ values for protonated cytosine, adenine, and guanine are, respectively, equal to 4.4, 4.1, and 3.3 [26,27,28]. Monoanion formation in the deprotonation of the neutral form of any of the compounds in Scheme 1 is not possible in acid media, that is, the ionization pK_a_ > 7 [25,28,29,30,31]. In line with these literature data, under the experimental conditions of the present investigation, the bases of Scheme 1 are neutral or cationic species.

## 2. Results and Discussion

None of the compounds studied in this work absorbs in the visible region of the electromagnetic spectrum. Below 400 nm, aqueous solutions of urea or TU are transparent in the concentration range used in this work. The spectral characteristics of the compounds shown in Scheme 1 are listed in Table 1 (Figure S1 of Supplementary Information shows the spectra). The absorption band of uracil at 259 nm remains unchanged in acetic acid (1.74 mM). The substitution of an exocyclic oxygen atom by a sulfur atom in 2TU or 4TU shifts to the red the maximum wavelength absorption; thus, 2TU shows two prominent absorption peaks due to π→π^*^ transitions, while 4TU exhibits a strong absorption band representing π→π^*^ transition and a weaker one at shorter wavelength, 242 nm, and no changes have been observed in mild acid medium. By contrast, the spectrum of cytosine changes from neutral to mild acid medium, and two clear isosbestic points were drawn attributed to the protonation at N_(3)_ site since the pK_a1_ is around 4.4. In neutral medium, adenine shows a broad band at 261 nm that, in 10.4 mM of acetic acid, λ_max_ shifts to 263 nm, which is consistent with protonation at N_(1)_H^+^ whose equilibrium step has a pK_a1_ = 4.1 [27,28]. Guanine solutions were prepared from the hydrochloric salt; either in neutral or in 10.4 mM acetic acid–water solution, the spectrum shows two absorption bands, that suggest the coexistence of two tautomers. In this respect, the free energy in solution of the two tautomers with the hydrogen bound to N_(7)_ or N_(9)_ differs only by 1 kcal/mol and their populations are very similar [27].

### 2.1. Urea and Thiourea

**Figure 1:**
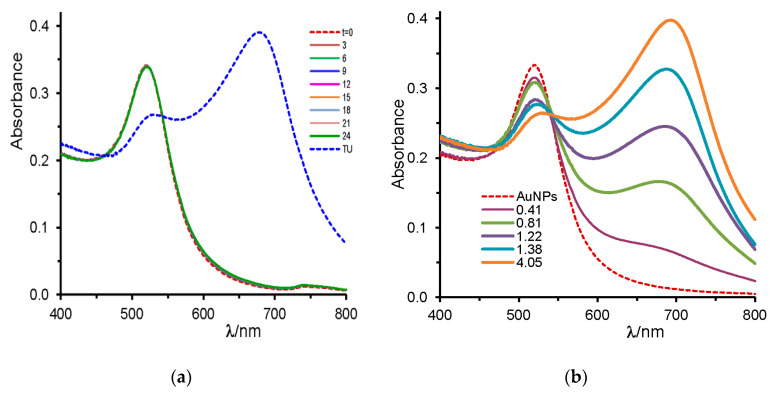
(**a**) Visible spectrum of 0.78 nM gold nanoparticles (AuNPs; dashed red line), and in the presence of 2.4 mM urea recorded at 3 min intervals over a 24 min period; the dashed blue line shows the spectra after adding 30 µL of thiourea solution to get [TU] = 8.1 µM; (**b**) effect of TU concentration (see insert: [TU] = 0.41; 0.81; 1.22; 1.38, and 4.05 μM) on the surface plasmon resonance (SPR) band of AuNPs.

Addition of urea at concentration 2.5 mM to a colloidal gold solution does not induce any change on its UV–Vis spectrum, neither in neutral nor in 10 mM acetic acid (Figure 1a); the presence of 0.10 mM Mn^+2^ ions (MnCl_2_) do not affect the spectrum. By contrast, the addition of a low thiourea concentration shifts the SPR band of gold hydrosol to 680 nm, and the solution color immediately changes from red to blue. The dashed blue curve on Figure 1a was recorded just after the addition of thiourea to the urea–AuNPs mixture, and the same effect was observed in the absence or urea (Figure 1b). TU adsorbs to the gold surface by displacing the weakly adsorbed citrate molecules and promotes the aggregation of NPs. This observation evidences that the adsorption of urea over the gold surface is negligible.

**Figure 2:**
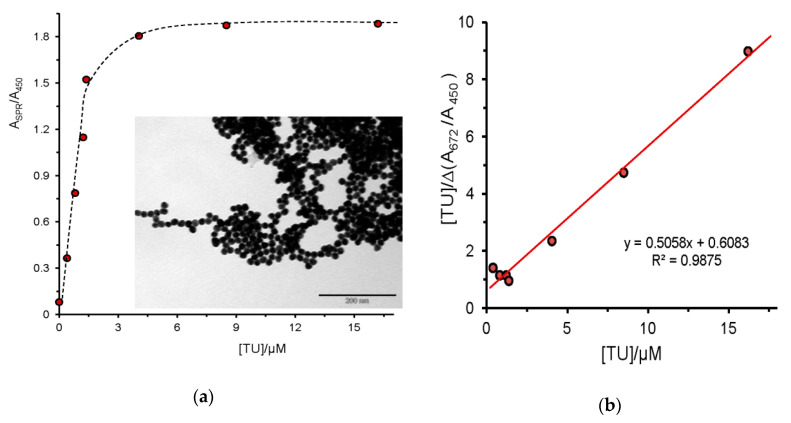
(**a**) Variation of the ratio of absorbance readings at 672 nm and 450 nm as a function of [TU], the insert shows the TEM image of AuNPs in the presence of 8.4 μM thiourea at 200 nm scale; (**b**) modified Benesi–Hildebrand plot for the absorbance ratio measured at A_672_/A_450_ as a function of TU concentration ([TU] = 0.4 to 16 μM).

A [TU] = 0.4 µM suffices to change the AuNPs spectrum; the effect increases with the TU concentration, Figure 1b. The plot of the absorbance ratio measured at 672 and 450 nm shows a strong increase with saturation level attained at approximately 4.0 µM, Figure 2a, when there are an average number of 5100 molecules of TU per nanoparticle. Taking into account the available surface of an AuNP of diameter 15.7 nm and the van der Waals radii of sulfur atoms (r_vdw_ = 0.18 nm), one estimates near 70% coverage of gold surface by TU molecules, which can be considered as a dense packing in a monolayer. On the other hand, the small change observed at the lowest [TU] resulted strongly increased in the presence of acetic acid (see Figure S2 of SI).

In order to quantify the interaction between thiourea (TU) and AuNPs, the modified Benesi–Hildebrand equation (double reciprocal plot or the Hanes plot in enzyme kinetics) [32] in the form of Equation (1) has been used, where R_e_ and R_o_ are the absorbance ratios (A_672_/A_450_) measured at the equilibrium (AuNPs/TU) and in the absence of TU, respectively. The equation works under the experimental conditions of [TU] >> [AuNPs]. Figure 2b shows the corresponding linear plot. From the fit, the average value of K_b_ was (8.3 ± 0.4) × 10^5^ M^−1^, which corresponds to an apparent free energy value of TU chemisorption of ∆G° = −33.8 kJ·mol^−1^; in addition, the slope of the corresponding plot yields a value of R_e_ = 1.977, which agrees with the experimental value. It is important to note that the λ_max_ shift due to the presence of TU is due to two inherent processes, thiourea adsorption over the gold surface and nanoparticles aggregation; then, the obtained binding free energy corresponds to the sum of both closely related processes and, consequently, one must refer to apparent free energy.

(1) $$\mathrm{AuNPs}+\mathrm{TU}\;\overset{K_{b}}{\Leftrightarrow }\;\mathrm{TU}\cdot \mathrm{NPs}\;;\;\frac{\left[\mathrm{TU}\right]}{R_{e}-R_{o}}=\frac{1}{R_{e}}\left(\left[\mathrm{TU}\right]+\frac{1}{K_{b}}\right)$$

**Scheme 2:**
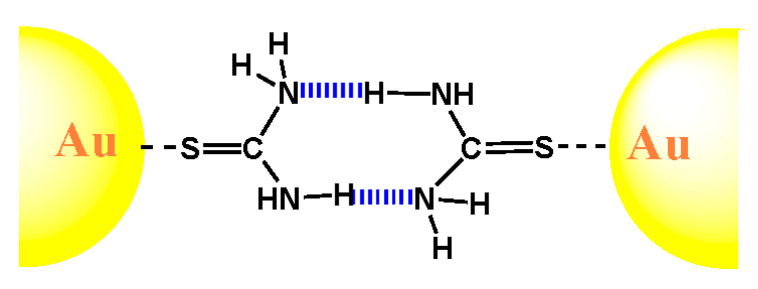
Cartoon to illustrate the TU binding mode to a gold surface and H-bonding interactions between two adjacent nanoparticles.

The large red-shift of the SPR band induced by TU is due to AuNPs assembly, as it is shown in the corresponding TEM microgram of the insert of Figure 2a. The sulfur of TU tends to really bind to the surface of colloidal gold particles via chemisorption-type interactions; at the same time, the amine groups of TU molecule from one NP can form H-bonds with the same group of an adjacent NP. Thus, the H-bonding network can act as cross-linking agent for pairs of TU-coated AuNPs, thereby inducing their aggregation. Scheme 2 illustrates the process.

### 2.2. Uracil, 2-Thiouracil, and 4-Thiouracil

**Figure 3:**
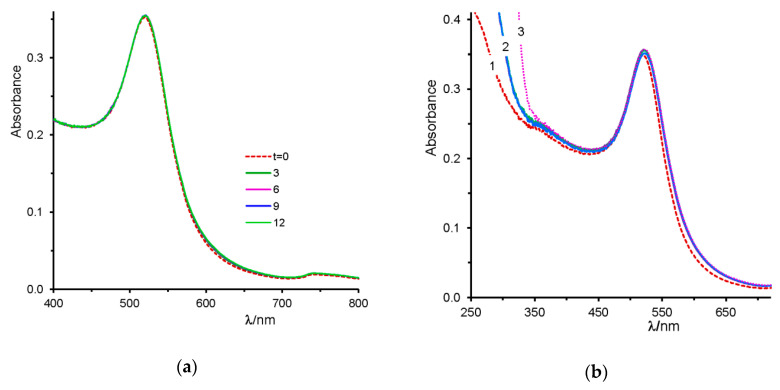
Spectrum of AuNPs solution (dashed line) in the presence of (**a**) 0.196 mM uracil recorded over 12 min at 3 min intervals, and of (**b**) 9.34 µM 2-thiouracil (curve 2 repeated for six cycles at 3.5 min intervals) and 156 µM (curve 3, which matches curve 2 above 450 nm).

Figure 3a,b shows the spectra of gold nanoparticle dispersions in the presence of uracil and 2-thiouracil, respectively. The spectrum in the presence of 2TU was recorded at two concentration values, 9.34 and 156 µM, and no color change was observed, that is, the colloidal solution is stable over a wide range concentration of either U or 2TU. However, noticeable differences can clearly be appreciated: the λ_max_ = 520 nm of the SPR band observed with uracil shifts to 523 nm in the presence of 2TU, which evidences the formation of sTable 2TU-capped gold nanoparticles. We have synthesized 2TU-functionalized AuNPs following a similar procedure to that of citrate capped gold nanoparticles, and recently these AuNPs were used in the colorimetric detection of uric acid [33]. Additional evidences in favor of the formation of 2TU-functionalized gold NPs are given in the next section.

**Figure 4:**
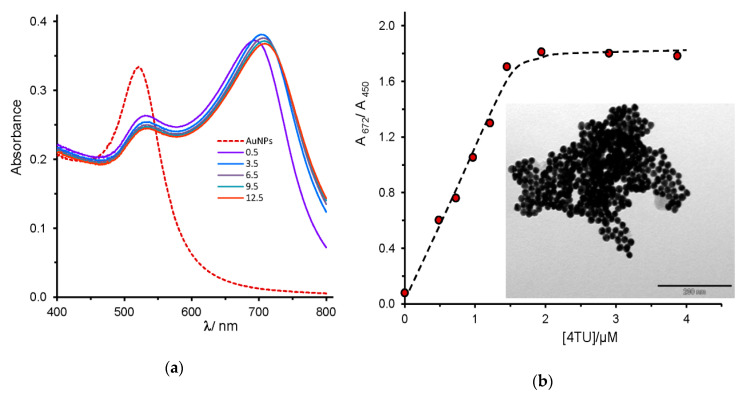
(**a**) Spectrum of AuNPs dispersion (dashed line) in the presence of 25.3 µM 4-thiouracil, recorded over 12.5 min at 3 min intervals; (**b**) ratio of the absorbance readings at 672 and 450 nm as a function of [4TU]; dashed line to guide the eye; the insert shows the typical TEM image of 4TU-mediated assembly of AuNPs.

By contrast, in the presence of 25.3 µM 4-thiouracil, the intensity of the plasmon resonance band at 520 nm suddenly decreases, and a new plasmon band at ~700 nm appears, see Figure 4a, indicating the assembly of gold nanoparticles. The SPR band red-shift was completed in less than 4 min, and subsequently starts to decrease slowly due the precipitation of NPs. The insert of Figure 4b shows the TEM image of AuNPs assembly promoted by 4TU. Like it has been observed with thiourea, increasing amounts of 4-thiouracil accelerates the NPs assembly process, as well as the extension of aggregation. Figure 4b displays the variation of the ratio of absorbance readings at 672 and 450 nm as a function of 4TU concentration. The saturation level was obtained at approximately 2 µM 4TU when the [AuNPs] = 0.845 nM, which means an average number of 2300 molecules of 4TU per nanoparticle. The theoretical maximum number of 4TU molecules attached to a nanoparticle can be estimated from the optimized structure of the 4TU molecule (see Figure S3 of SI), and its cross-section in contact with the surface of a nanoparticle. We assume the NP surface is equal to that of a sphere of diameter 15.7 nm, i.e., 770 nm^2^. The cross-section area of 4TU, considered as an ellipsoid, was estimated as being 0.15 nm^2^; then, from these figures, the theoretical full surface coverage of a nanoparticle requires 5250 molecules of 4TU. Therefore, the degree of surface coverage is estimated as 44%, which should be considered as a submonolayer.

Following the same approach as with thiourea, the fit process of the absorbance ratio values (A_672_/A_450_), determined as a function of 4TU concentration, to Equation (1) gives an average value of the binding constant of 4TU over the gold surface K_b_ = (11.1 ± 1.5) × 10^5^ M^−1^, that is, comparable to the binding constant obtained for thiourea, a fact that indicates the same binding mode as S atoms to the gold surface, Scheme 2.

**Scheme 3:**
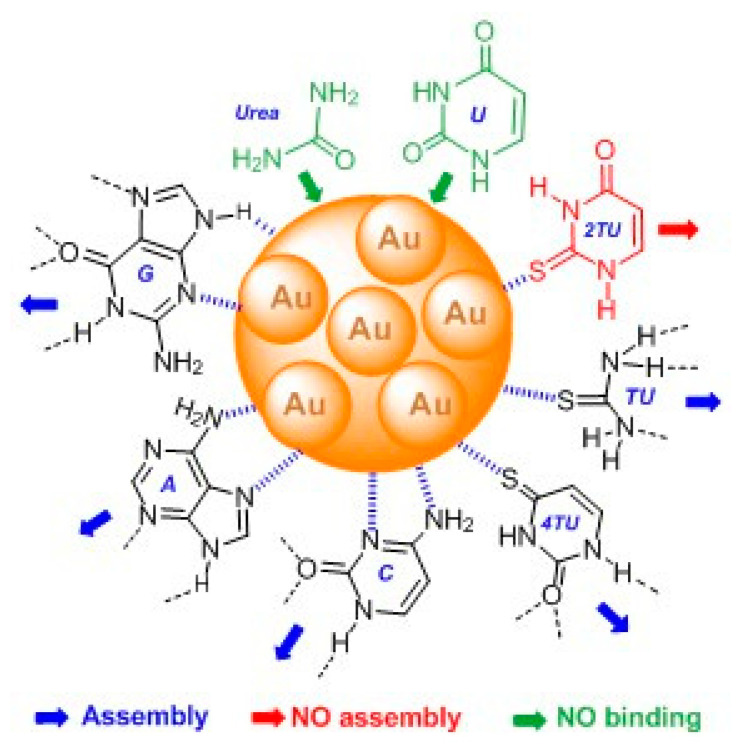
Schematic representation of the binding modes of the studied DNA bases and related compounds: Black structures represent the bases that mediate AuNPs aggregation; the red compound coats the gold surface, but does not induce aggregation, and green compounds neither bind to the gold surface nor induce NPs assembly.

The aggregation of gold NPs due to the interparticle interaction mediated by 4TU can be stopped when 2TU is added to the sample mixture. By contrast, when 25.3 µM of 4-thiouracil (added volume 30 µL) is poured into 0.97 mL of AuNPs aqueous solution, that is, 93.9 µM in 2-thiouracil, the aggregation of AuNPs does not occur (Figure S4a). In the same manner, when 0.30 mL of 2.60 nM AuNPs is poured into 0.70 mL of an aqueous solution, that is, 93.4 μM in each compound (2-thiouracil and 4-thiouracil), the assembly of AuNPs was not observed (Figure S4b). These observations suggest that 4TU molecules adsorbed on the gold surface are not displaced by 2TU molecules, but the binding of 2TU to the gold surface is stronger than that of 4TU. The binding mode of the –C=S group, suggested in Scheme 3, clarifies the absence of AuNPs aggregation.

### 2.3. Cytosine, Adenine, and Guanine

These DNA bases do not have sulfur atoms; nevertheless, all the three bases mediate assembly of gold nanoparticles at micromolar concentration. The process of interparticle interactions is slower than that induced by the sulfur-containing compounds, such as thiourea or 4-thiouracil.

**Figure 5:**
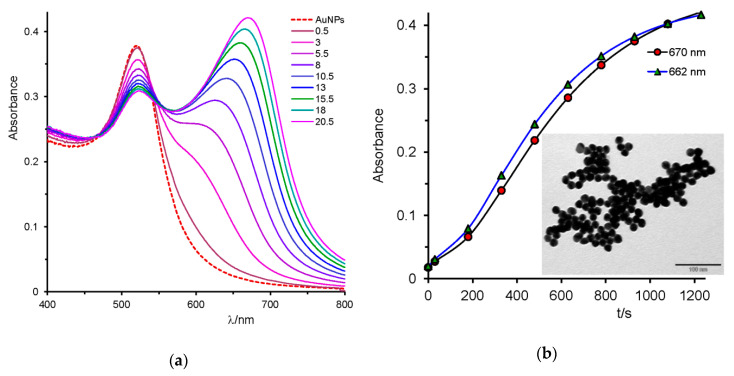
(**a**) Evolution of the SPR band of gold nanoparticles in the presence of 11.3 µM cytosine recorded over 20.5 min at 2.5 min intervals (dashed spectrum is only NPs); (**b**) plot of the absorbance readings at (●) 670 nm and (▲) 662 nm as a function of time; the insert shown a TEM image of gold nanoparticles assembly mediated by cytosine.

Cytosine is the uracil derivative in which the exocyclic oxygen is replaced by a primary amine group. As we have indicated in the previous section, we have not observed the aggregation of AuNPs mediated by uracil under different experimental conditions. On the contrary, cytosine strongly interacts with AuNPs. Figure 5a shows the evolution of the SPR band of AuNPs in the presence of 11.3 µM cytosine; the optical intensity at 520 nm slightly decreases, whereas a new plasmon band at higher wavelength increased exponentially with time. The corresponding absorbance readings at 662 or 670 nm are plotted in Figure 5b as a function of time. Good first-order kinetics were observed (the first two points taken in the absence of cytosine and at 20 s after the addition of cytosine were not included). The rate constant for the assembly process was determined as k = (2.0 ± 0.1) × 10^−3^ s^−1^ at [C] = 11.3 µM and k = (1.90 ± 0.04) × 10^−3^ s^−1^ at [C] = 5.75 µM; nevertheless, the millimolar cytosine concentration causes reactions too fast to follow by conventional methods. The presence of small quantities of acetic acid accelerates the assembly process, e.g., k = (3.75 ± 0.02) × 10^−3^ s^−1^ at [C] = 9.5 µM and [acetic acid] = 4.2 mM. In mild acid medium, cytosine may be protonated on N_(3)_ to yield the corresponding cation (pK_a1_ = 4.4), which favors the interaction with the negatively charged citrate-capped gold nanoparticles.

Given that the only difference between uracil and cytosine is the amine group in the latter, this group must adsorb to the gold surface. We assumed a binding mode of the type shown in Scheme 3, where both the amine group and the N_(3)_ are bonded to gold surface, in agreement with FTIR results by Borse et al. [8], according to them, the change of the stretching bonds C_(4)_–NH_2_ and C_(4)_–N_(3)_ of cytosine are the most affected by the presence of AuNPs. Ab initio calculations, supported by spectroscopic experiments, point to metal surface adsorption via –NH_2_, where the N atom shows sp^3^ hybridization [34].

On the other hand, the presence of cytosine does not induce the aggregation of 2-thiouracil-capped gold nanoparticles; in fact, addition of 34 µM cytosine into the AuNPs solution containing 63 µM 2TU does not induce any observable effect; no spectral signature for aggregated nanoparticles was observed, and higher cytosine concentration behaves equally. However, with the addition of both nucleobases (cytosine and 2-thiouracil) in the reverse order, the aggregation process was firstly induced by cytosine and then was stopped after the addition of 2TU, but it was not reverted. This observation supports the proposed binding mode; otherwise, the NPs assembly should be reverted by 2TU if one takes into account the stronger Au–S bond than that of –N–Au, but perhaps weaker than two –N–Au bonds.

**Figure 6:**
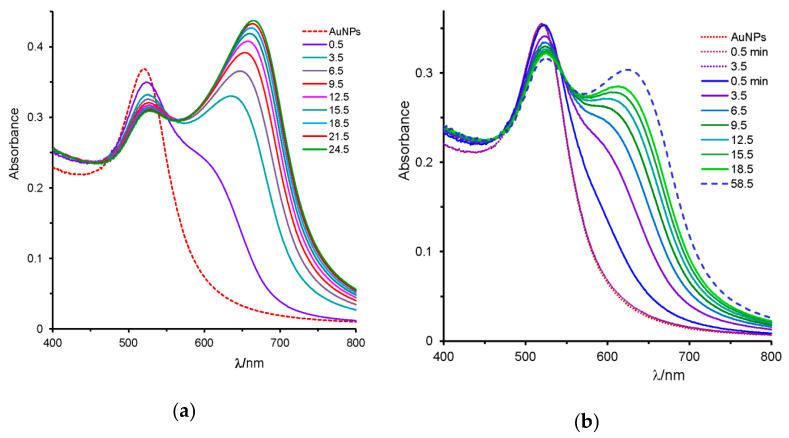
Evolution spectra of the SPR band of AuNPs in the presence of (**a**) 1.55 µM adenine recorded over 24.5 min at 3 min intervals; (dashed curve) only nanoparticles (NPs); (**b**) dotted curves correspond to AuNPs in the absence and in the presence of [Mn^+2^] = 0.046 mM after 3.5 min; solid curves were recorded at 3 min intervals after adding 8.4 µM guanine hydrochloride; and dashed curves are 40 min after the latter.

Finally, Figure 6a,b shows the representative spectra monitoring the evolution of the SPR band of gold nanoparticles upon the addition of either 1.55 µM adenine or 8.4 µM guanine, respectively. The surface plasmon resonance band of gold NPs shifts to longer wavelengths due to the aggregation of AuNPs, with maxima at 663 or 620 nm in the presence of adenine or guanine, respectively. The H-bonding interactions between the DNA bases adsorbed to the gold surface of adjacent nanoparticles and possible π−π and hydrophobic interactions can essentially decrease the interparticle distance and induce color change due to aggregation. The rate of the nanoparticle assembly process increases with the nucleobase concentration; the presence of a small concentration of either acetic acid or Mn^+2^ ions, under conditions of stable AuNPs, accelerates the process [21]. The increase of adenine concentration favors interparticle interactions and the aggregation process is faster (Figure S5a). The process follows first-order kinetics, with the rate constant determined for [A] = 1.55 µM as k = (2.60 ± 0.03) × 10^−3^ s^−1^, whereas with [A] = 38.8 µM, it increases to k = (5.75 ± 0.01) × 10^−3^ s^−1^, and in mild acid medium of 1.6 mM acetic acid, the process is too fast to monitor by conventional techniques.

We assumed that adenine adsorbs to the gold surface by chemisorption-type interactions through C_(6)_–NH_2_ and –N_(7)_, i.e., by amine group and N–imidazole interactions. This orientation leaves both the N_(3)_ and N_(9)_H facing outward from the nanoparticle surface, which can act as bridging linkers between one NP and another due to strong H-bonding interactions. The effect of acetic acid can be understood by considering the protonation of adenine (N_(1)_H^+^ of pK_a1_ ~ 4.1); the resulting cationic substrate interacts faster with the negatively charged citrate monolayer that stabilizes nanoparticles.

However, the orientation of the base adsorbed to the gold surface is still under debate; in fact, different modes with the base ring perpendicular to the metal surface, parallel, or tilted have been proposed, which can be combined with different sites of interaction; e.g., for C, N_(3)_ nitrogen as well as the keto oxygen -C_(2)_=O and N_(3)_ nitrogen, exclusively, have been proposed. [34,35,36,37].

In the same manner, the process of AuNPs aggregation mediated by guanine follows first order kinetics (Figure S5b), with rate constants equal to k = (0.385 ± 0.002) × 10^−3^ s^−1^ at [G] = 5.6 µM; k = (0.680 ± 0.006) × 10^−3^ s^−1^ at [G] = 8.4 µM, and k = (1.670 ± 0.004) × 10^−3^ s^−1^ at [G] = 8.4 µM and [Mn^+2^] = 0.046 mM. Clearly, guanine is much less effective than adenine or cytosine. This fact would suggest the interaction with gold surface in the mode represented by Scheme 3. This explains the efficiency of low [G] and the slower aggregation rate, because of the less feasibility of H-bonding network.

Table 2 summarizes all the observed effects upon the addition of nucleobases to a colloidal gold solution, either individually or in pairs of bases.

## 3. Materials and Methods

### 3.1. Materials

All the chemicals were of the maximum available purity and were used without further purification. Hydrogen tetrachloroaurate(III) trihydrate (HAuCl_4_ × 3H_2_O) and trisodium citrate (Na_3_C_6_H_5_O_7_·2H_2_O) were purchased from Alfa Aesar (Germany); uracil, cytosine, adenine, guanine, 2-thiouracil, and 4-thiouracil were Sigma products, and urea and thiourea were supplied by Merck (Madrid, Spain). The water used in all solutions was firstly deionized and subsequently distilled twice (the first distillation was over potassium permanganate).

### 3.2. Synthesis of AuNPs

Gold nanoparticles were prepared by reduction of 0.28 mM HAuCl_4_ with 1.12 mM of trisodium citrate during 20 min. Briefly 1.5 mL of a trisodium citrate solution was mixed with 50 mL of aqueous solution of hydrogen tetrachloroaurate at boiling temperature under vigorous stirring. Initially the mixture turns faintly blue, then suddenly changes into a wine-red color, indicating the formation of monodispersed citrated-capped gold nanoparticles. The transmission electron microscope (TEM) image shows that the gold NPs are nearly monodispersed and spherical in shape, with very few of triangular shape. The average size was d = 15.7 ± 1.3 nm (Figure S6). The SPR band was centered at 520 nm (A_520_ = 1.12 and A_450_ = 0.665), and the nanoparticles concentration resulted [NPs] = 2.60 nM.

### 3.3. Methods

UV–Visible spectra were recorded with a Kontron-Uvikon double beam spectrophotometer fitted with thermostated multicell holder. Spectra were collected over the range 200 to 800 nm using quartz cells of 1.4 mL volume. Stock solutions of the nucleobases were prepared in water. An aliquot of each of these solutions was added to the gold colloidal solution to give a total volume of 1 mL. In a typical experiment, 0.30 mL of the previously prepared AuNPs was diluted with water and the required volume of the solution base (urea, thiourea, uracil, etc.) to reach a final concentration of 0.78 nM (the citrate concentration was lower than 0.34 mM). At this concentration of NPs, the colloidal solution shows an absorption band at 520 nm (A ~ 0.35) due to SPR, which is the result of the collective oscillation of the conduction electrons across the NP as a consequence of the resonant excitation by the photons of the incident radiation.

In studying the kinetics of the evolution of the SPR band, the absorbance (A) versus time (t) were recorded and fit to the first-order integrated rate equation, A = A_∞_ − (A_∞_ − A_0_)·*exp* (−k × t). The non-linear regression analysis of A-t data gives k, A_0_, and A_∞_ as optimizable parameters, with k being the first order rate constant and A, A_0_, and A_∞_, the absorbance values at times t, zero, and at the end of the reaction. All experiments were conducted at 25 °C.

The pH was measured with a Crison 2001 pH meter equipped with a GK2401B combined glass electrode and calibrated using commercial buffers of pH 4.01, 7.02, and 9.26 (Crison, Barcelona, Spain).

Transmission electron microscopy (TEM) was carried out using a JEOL JEM 1010 electron microscope operating at an acceleration voltage of 100 kV and equipped with a Mega View III camera controlled with Analysis software. Samples of TEM analysis were prepared by placing drops of gold colloidal solutions onto a carbon-coated copper grid sample holder, followed by evaporation in air at room temperature. The reported TEM images are representative on the entire grid sample.

## 4. Conclusions

A nonselective method for detection of nucleobases and derivatives using AuNPs was reported. The simplicity, rapidity, and high sensitivity are remarkable advantages against the classical methods, which require long manipulating time. Urea and uracil do not induce any effect in the spectral response of the AuNPs solution. The spectral signature observed with 2-thiouracil indicates the formation of sTable 2TU-capped gold nanoparticles. Contrarily, the color of the gold colloidal solution instantaneously changes from red to blue in the presence of micromolar concentrations of thiourea, 4-thiouracil, cytosine, adenine, or guanine. These bases promote the aggregation of AuNPs because of the interparticle interaction by H-bonding between the bases adsorbed to gold surface of adjacent nanoparticles. The aggregation process follows first-order kinetics and is accelerated in mild acid medium or in the presence of small amounts of manganese ions. Nevertheless, dispersed 2TU-capped gold nanoparticles are completely stable even in the presence of the other bases studied in this work. The results can be extended to applications in the field of DNA nanotechnology.

## Figures and Tables

**Table 1:** UV–Vis spectral characteristics of the bases dissolved in water and values of the pK_a_ corresponding to the protonated base, ${\mathrm{BaseH}}^{+}\;⇌\mathrm{Base}+H^{+},{\;K}_{a1}$.

Base ^(a)^	λ_1_/nm	Ɛ_1_/mol^−1^dm^3^s^−1^	λ_2_/nm	Ɛ_2_/mol^−1^dm^3^s^−1^	pK_a1_
urea	---	---	---	---	0.05
thiourea (TU)	---	---	---	---	−0.90
uracil (U)	259	8150	---	---	<0.5
2-thiouracil (2TU)	212	17,000	270	14,000	---
4-thiouracil (4TU)	242	4100	328	19,000	---
cytosine (C)	268 (neutral)	7050	275 (acid) ^(b)^	9750	4.4
adenine (A)	261	13,200	263 (acid) ^(c)^	12,900	4.1
guanine (G)	246	6300	276	4600	3.3

^(a)^ the concentration of the base varies between 42 µM (4TU) and 77.6 µM (A), see Figure S1; ^(b)^ acetic acid 1.74 mM; ^(c)^10 mM acetic acid.

**Table 2:** Observed effect upon addition of base 1 at the concentration value in parentheses, and subsequently base 2, on an aqueous colloidal gold solution.

Base 1 (c/μM)	Base 2 (c/μM)	λ_max_ (SPR band)/nm	Color	Effect
urea (2500)	---	520	red	−
thiourea, TU (0.4 to 8.1)	---	670	blue	+
urea (2500)	thiourea, TU (8.1)	670	blue	+
uracil, U (196)	---	520	red	**−**
2-thiouracil, 2TU (9.34)	---	523	red	functionalized
4-thiouracil, 4TU (25.3)	---	672	blue	+
2TU (94)	4TU (94)	523	red	−
2TU (94) + 4TU (94)	AuNPs addition	523	red	functionalized
cytosine, C (11.3)	---	670	blue	+
2TU (63)	C (34)	523	red	**−**
C (34)	2TU (63)	red-shift	--	stopped
adenine, A (1.55)	---	663	blue	+
guanine, G (8.4)	---	620	blue	+

## Supplementary Materials

The following are available online, Figures S1–S6, showing the spectra in aqueous neutral or acid media of nucleobases, the effect of either acetic-acid or Mn^+2^ ions in AuNPs self-assembly rate, as well as the effect of nucleobase concentration, and the histogram of NPs size distribution.
