# Supplementary material for: Gold Nanoparticles as Colorimetric Sensors for the Detection of DNA Bases and Related Compounds
Source: Molecules. 2020 Jun 23;25(12):2890. doi: 10.3390/molecules25122890 (PMC7356728; doi:10.3390/molecules25122890)
Supplement: Supplementary file 1 [file molecules-25-02890-s001.pdf]

SUPLEMENTARY INFORMATION

Gold nanoparticles as colorimetric sensors for the detection of DNA bases and related compounds

Emilia Iglesias

Departamento de Química. Facultad de Ciencias. Campus A Zapateira. Universidad de La Coruña. 15008-La Coruña. SPAIN. emilia.iglesias@udc.es

Figure S1: The spectra of the compounds studied in both neutral and mild acid aqueous solution.

Figure S2: Experiments to compare the effect of small amounts of acetic acid in the gold nanoparticles aggregation mediated by thiourea.

Figure S3: Optimized structures of thiourea and 4-thiouracil.

Figure S4: Experiments to demonstrate the effect of order addition of 2-thiouracil, 4-thiouracil, and AuNPs solution in the mixture.

Figure S5: First-order plot of absorbance *versus* time corresponding to AuNPs aggregation process mediated by adenine and guanise

Figure S6: Histogram of size distribution of synthesized nanoparticles.

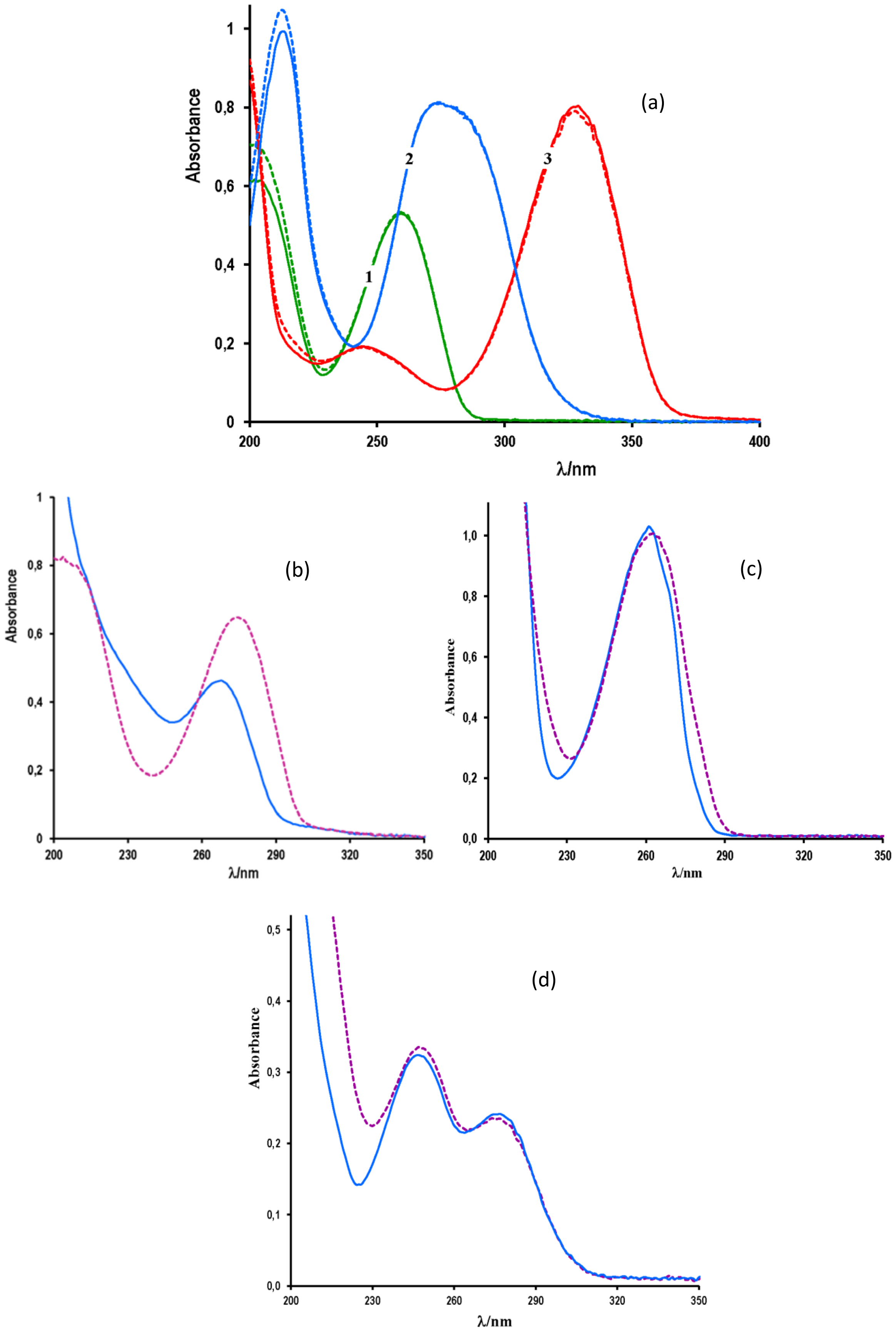

Figure S1. UV-Vis absorption spectrum **(a)**of (1)Uracil 65 μM; (2)2-thiouracil 58 μM, and (3)4-thiouracil 42 μM, in water and in 1.74 mM acetic acid (*dashed line*); **(b)**cytosine 65 μM in water and in 1.74 mM acetic acid (*dashed line*), and **(c)**adenine 77.6 μM, in water and in 10.4 mM acetic acid (*dashed line*); **(d)**guanine 50 μM in water and in 10.4 mM acetic acid (*dashed line).*

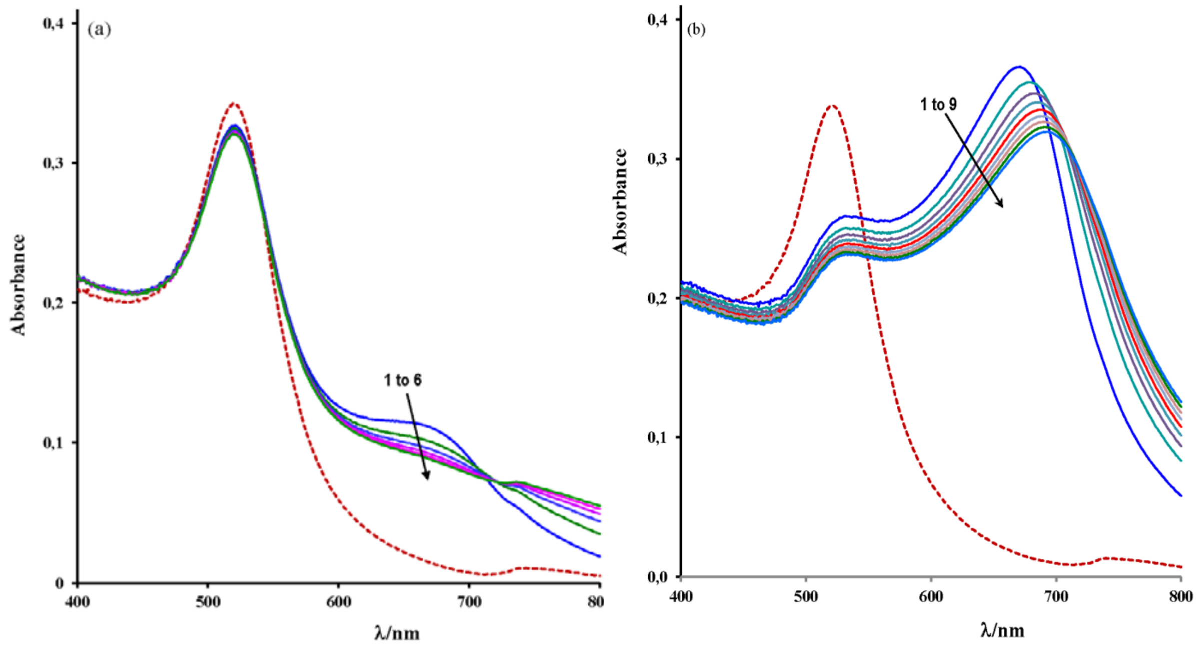

Figure S2. The absorption spectra evolution of AuNPs solution in the presence of 1.12 μM of thiourea (a)in the absence of acetic acid, and (b)at 1.6 mM of acetic acid; (- - -) only AuNPs; (scans 1 to 6 or 1 to 9) at 3 min interval.

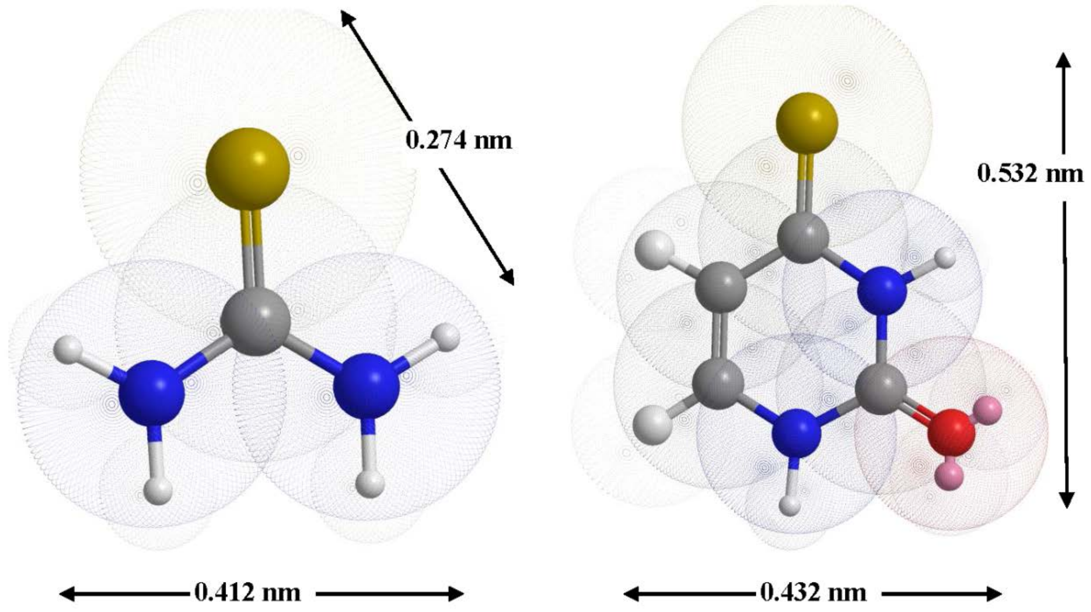

Figure S3. Cross-section dimensions of the optimized structures of thiourea and 4 thiouracil

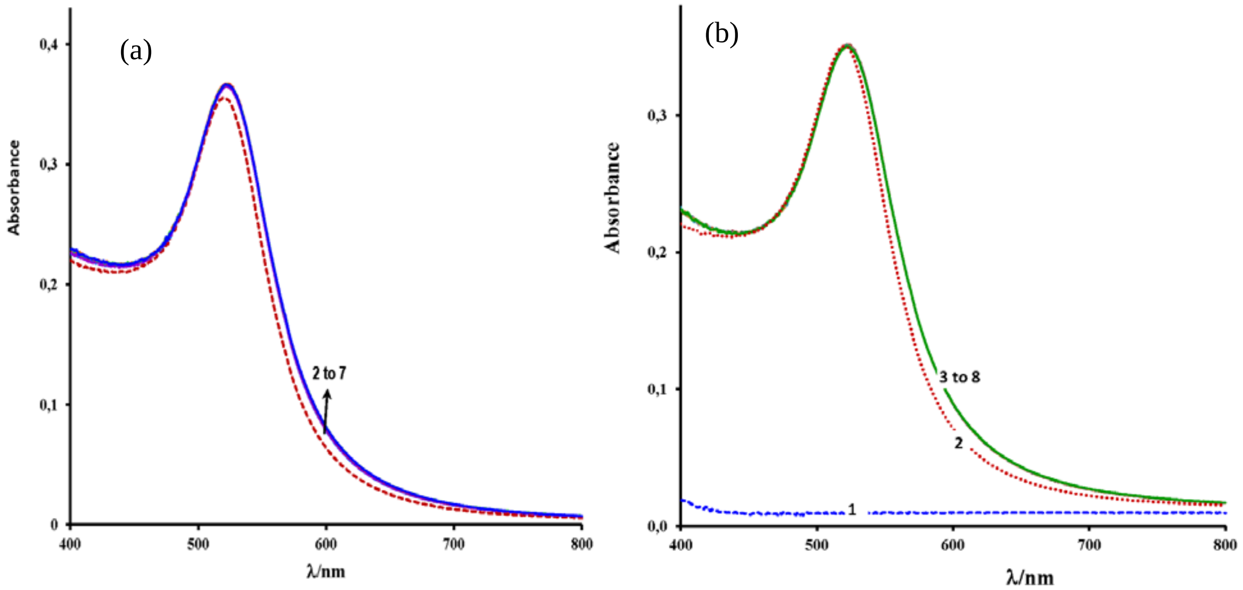

Figure S4. UV-Vis spectrum of gold nanoparticles solution (- - -) and in the presence of (a)93,9 μM of 2TU recorded at 3 min interval (curves 2 to 6), and after the addition of 25.3 μM of 4-thiouracil (curve 7); no changes have been observed, and (b)93.9 μM of both 2TU and 4TU but in the absence of AuNPs (*dashel line 1*); only AuNPs in the absence of 2TU and 4TU (*dotted line 2*), and with the three additives: 2TU, 4TU and AuNPs added in this order (*curves 3 to 8* recorded at 3 min interval). The different additives were added into the same sample successively, the effect of volume increase has been corrected.

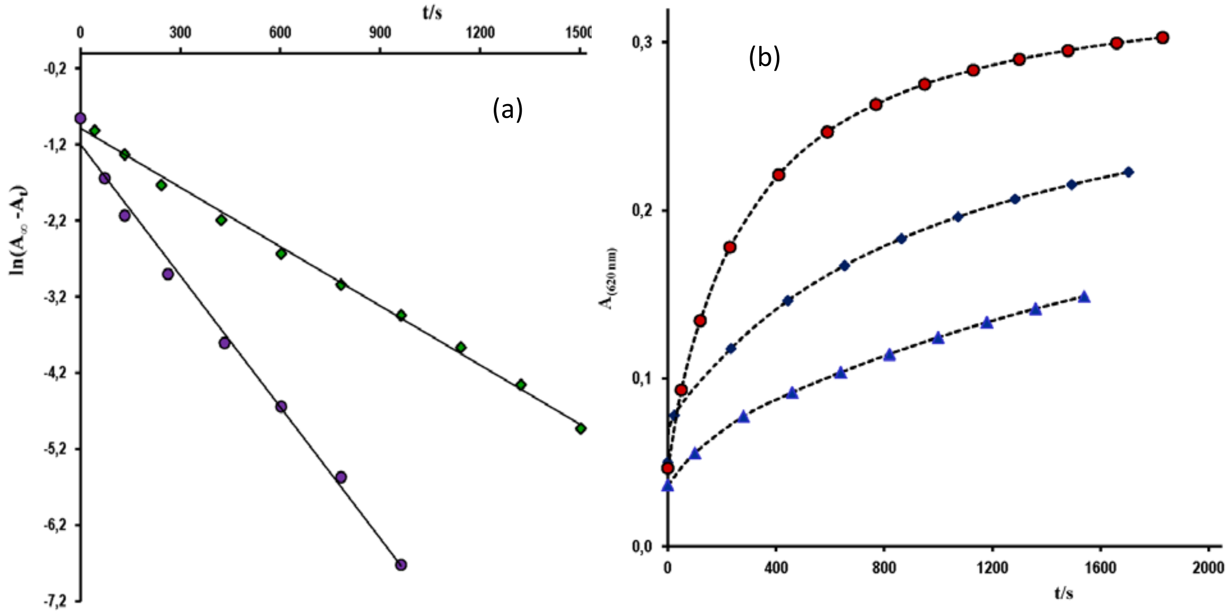

Figure S5. (a) Linear form of the first-order rate equation $\{A_t=A_\infty-(A_\infty-A_o)exp(-k\cdot t)\}$ for the absorbance increase at 670 nm as a function of time corresponding to the SPR band of gold nanoparticles solution in the presence of adenine at (◆)[A]=1.55 μM, and (•)[A]=38.8 μM. (b) Increase absorbance at 620 nm due to the evolution of the SPR band of gold NPs in the presence of guanine at(▲)[G]=5.6 μM; (◆)[G]=8.4 μM, and(•)[G]=8.4 μM, $[Mn^{+2}]$= 0.046 mM.

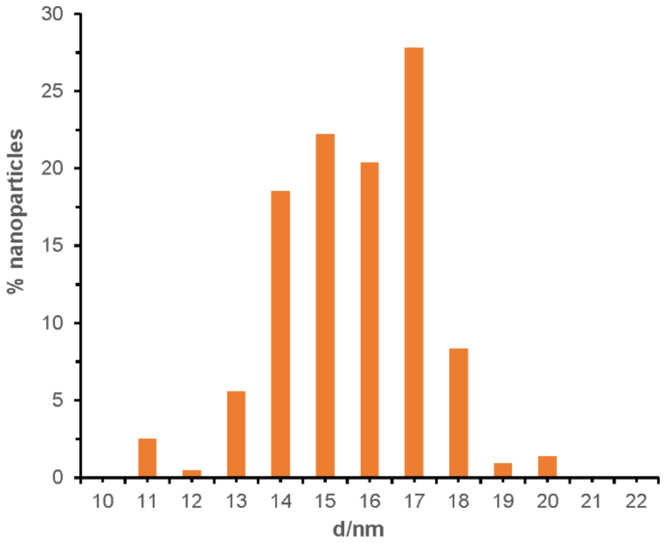

Figure S6. Size distributions of synthesized gold nanoparticles
